# Round the Hospitals

**Published:** 1917-10-27

**Authors:** 


### ROUND THE HOSPITALS.

"Our Day," October 18, on behalf of the British Red Cross Society and the Order of St. John, provided a record collection, for the total already amounts to over £1,000,000. The following notable gifts were announced:—His Majesty the King £10,000, against £5,000 the previous year, given in appreciation of the great work of mercy carried out by the united efforts of the Red Cross organisers, which has been deepened, if that were possible, by the constant opportunities during the past twelve months which His Majesty has had of witnessing afresh, both at home and in Flanders, their magnificent services. Her Majesty generously gave £1,000, and H.R.H. the Prince of Wales £3,000. The American Red Cross sent a subscription of £200,000—£50,000 for relief and comforts for the sick and wounded in hospitals, casualty clearing-stations, and on lines of communication in territories where the British forces are fighting; £50,000 for the maintenance of British Red Cross auxiliary hospitals and convalescent homes in England; and £100,000 for institutions in Great Britain for orthopædic and facial treatment, and for general restorative work for disabled British soldiers. The American gift was sent with an expression of the hope that it would be accepted as an earnest of the desire of the people of the United States to begin to take their share in the burden of the war which the British Forces have waged for over three years on behalf of the whole civilised world.

The action of the British Women's Hospital organisation in issuing their manifesto for the Nation's Fund for Nurses, with the combined object of placing the College of Nursing upon a sound financial basis, capable of establishing with energy and completeness a recognised standard of education for nurses, providing the nursing profession through the co-operation of British nurses with faultless machinery to meet existing difficulties, and supplying a perfected organisation under a central authority, empowered to direct, control, and mobilise it, has been welcomed by all classes of people in the British Islands. It required a stout heart, a noble courage, and high administrative gifts to launch a great scheme like this, and the step taken will win for the British Women's Hospital authorities the welcome and gratitude of British people, and bring them, as we hope and believe, lasting fame. The enemy has tried for months to sow tares in the nursing field, but its methods and objects have been made so clearly manifest by the action taken by the Editor of the *Sunday Times* in opening his columns for months to enable this to be done, that a renewal of similar efforts may properly receive the W.P.B. seal of other editors up and down the country. Such a result would be poetic justice, for the British Women's Hospital authorities have no axes to grind, and the enemy's axe must already be worn out by its immoderate and excessive use since the College of Nursing had its birth.

The directing minds and supporters of the British Women's Hospital deserve a splendid and overwhelming success for their appeal as a thank-offering from the British Empire to British Nurses. It is fitting that they should in this way associate themselves with Florence Nightingale, for they have already shown themselves to be possessed of some of her genius, her determination, and her sympathetic mind. This committee, as Sir Frederick Treves testifies, has already provided all the money needed to erect on Richmond Hill a permanent memorial of the Great War in the form of a Home for the disabled sailor and soldier. An attractive feature of their present effort is the fact that the Tribute Fund will be administered jointly by the Council of the College of Nursing and the Committee of the British Women's Hospital. We have no doubt that the millionaire's cheque and the widow's mite will help alike to further this splendid work.

A public meeting for nurses, who are cordially invited, will be held in the Examination Hall of the Royal College of Physicians, 6 Kildare Street, Dublin, on Monday, 29th instant, at 5 p.m. Dr. Peacocke, chairman of the Irish Board, will preside, and Miss Matheson, secretary to that Board, will be the speaker. After her address the meeting will be opened to a discussion of the question of the economic status of the Irish nurse. It is

**ROUND THE HOSPITALS**—(*continued*).

hoped that the nurses attending will come prepared with suggestions and back them by the further expression of their opinions on this subject.

We have received the following copy of a letter dated October 23, addressed to the secretary of the Royal British Nurses' Association by Miss M. S. Rundle, secretary of the College of Nursing:—

"Referring to a letter addressed to the nursing press, dated October 16, 1917, and signed by the hon. secretaries and secretary of the Royal British Nurses' Association, I would like to say that I regret that anything I have said concerning the amalgamation of the College of Nursing, Limited, with the Royal British Nurses' Association should have been regarded as 'misleading and inaccurate.' The suggestion of amalgamation came about as the result of pourparlers between members of the two bodies, and I am not prepared to admit that it was first mentioned by the promoters of the College, but I fully admit that the first official advance came from the College, as the younger Association. This is but another proof of the policy of the College, which has always been to endeavour to effect unity in the profession, for as the hon. secretary of your Association has himself said: 'Two bodies united and working for a common object are of greater driving force than half a dozen separate bodies with the same aims, but working independently on different lines. Union is strength.' The position of the two Associations in relation to the actual amalgamation, viz., that the College was to amalgamate with the Royal British Nurses' Association, and not the Association with the College, has been made common knowledge from the first and is indisputable, and if reference is made to the report of my address it will be seen that I used the word 'amalgamation' as synonymous with 'affiliation,' an inappropriate word which was a verbal slip on the part of the speaker, and certainly was never meant to have the misleading significance attached to it in the letter."

On October 24, at the Nightingale Home, Miss Amy Hughes presented the Nightingale Medals awarded this year to the successful nurse-competitors at St. Thomas's Hospital. Only the Nightingale nurses were present, and the occasion will be memorable in the history of the Home from the circumstance that this year not only the silver and bronze but the gold medal have been won and presented. Under Miss Lloyd Still's able direction continuous efforts have been made to increase the efficiency of the training of Nightingale nurses. The appointment of a sister-tutor has been fruitful in results for good, and has proved a most attractive feature to many who have high ambition to enter for training as a nurse under the most modern and up-to-date conditions, with the prospect of extending their education, improving their intellectual outlook, and qualifying for the higher posts in the nursing world. The Nightingale Medals, which were designed by the Countess Feodora Gleichen, the successful sculptor of the statue of Miss Nightingale erected at Derby, are singularly attractive and of real beauty. It is a matter of taste which metal has lent itself best to beautify the design; we are inclined to think the silver medal is on the whole the most remarkable in this respect. The fortunate recipients of the medals were:—Gold Medallist, Nurse Edith Mary Heale; Silver Medallist, Nurse Ekaterini Stefanos Servetopoúlos; Bronze Medallist, Nurse Theodora Meanie West-Watson.

St. Thomas's Hospital is fortunate in having on its staff some who have the artistic sense and whose judgment in the selection of designs may be trusted to be of the highest quality. Each medal on the obverse bears the head of Florence Nightingale, surrounded by the legend, "I have taught thee in the way of Wisdom," taken from the Book of Proverbs. For the reverse a Greek bas-relief now in the Terme Museum at Rome has been reproduced. It represents the Lady with the Lamp, or the Spirit of Nursing, keeping alive by her constant care the flickering flame of life. Balancing this design is a small cartouche on which is engraved the year of award. On the cartouche an owl is perched, the emblem of wisdom. The owl is traditionally connected with Florence Nightingale, who, when visiting the Acropolis in early life, received from some boys a baby owl which had fallen out of its nest in the Parthenon. Miss Nightingale brought this owl to England, where it long lived with her as a pet. We may have something more to say about the medals and their award on a future occasion.

Dr. C. R. Rutland, M.D. (Brussels), of 2 Weymouth Court, Weymouth Street, W. 1, sends a sample of a bandage fastener he has devised, which he claims combines security with extreme rapidity in application. The Ministry of Munitions have granted him a priority order for steel wire to produce 150,000 pins for trial purposes, which puts him in a position to offer small supplies of these pins to Red Cross Hospitals, surgeons, nurses, and all who have wounded soldiers under their care. It is claimed further that this safety-pin obviates the serious disadvantages of the ordinary one in use, obviating the danger to the nurse of contracting septic fingers, and the loss of tightness due to the necessity for inserting the finger under the bandage when applying it. We shall be glad to have a report on this new safety-pin from any readers who may accept the doctor's invitation to test it in actual use.

The quality of leadership is one increasingly needed in the control and management of nursing affairs. Women workers, and amongst them the best of nurses, have shown such courage, enterprise, and devotion during the present terrible war that the people of all classes of the Allied nations feel towards them as they do towards the members of the R.A.M.C. and the practitioners who have given up everything to help our wounded warriors, and, with the orderlies and other non-combatants, by their bravery, self-sacrifice, and devotion, have won the recognition of all our fighting men, who feel

**ROUND THE HOSPITALS**—(*continued*).

that in this war the claim to honours and recognition extends equally to combatants and non-combatants alike, for all have been splendid and all have earned from the people in the Homeland, and the Nations they have served, everything of the best, for nothing can be too good for each and all of them. This is the feeling which is to be found in every part of the world where the Great War has been waged or is waging. To share the work and inspect every section of it is an inspiration, only limited in its extent by the knowledge of the observer who has had the privilege to face the risks and go through the battlefields, the hospitals, the clearing stations, advance posts, and out to the Front, with the doctors, nurses, and orderlies, whose duty it is to devote their whole energies to the collection, treatment, nursing, ambulance comforts, and recovery of the wounded and the sick.

---

A probationer nurse, shortly to complete her training, writes to thank "A Matron of Many Years' Experience" for her short article in The Hospital of October 13 on "The Responsibility of Matron and Nurse." She rejoices that "Matron" states plainly that in her view personal character is as essential as professional efficiency. The nurse in training recognises the advantage of the higher education for aspirants to the nursing profession, but maintains there is hope, and a career in nursing, for women of high intelligence, who may be less educated, but aspire to that position. Our correspondent has found that a happy life for the probationer during training greatly depends on the matron's attitude towards her. A matron who sympathises with the real difficulties of the junior members of the staff training under her control cannot fail to be happy. They will treasure her teachings, and absorb high qualities from the matron's example and character. They will carry her teachings into practice in their after-career, and so spread the fame of their *Alma Mater* and its responsible head. It may interest matrons generally to have this candid opinion from the ranks of those now undergoing training in this country.

---

A soldier lay dying of his wounds. As the doctor leaned over his bed the man whispered, "Hold my hand." A few seconds later he let go the doctor's hand, smiled an apology for the weakness of his request, and died. Little daily bedside scenes such as these mostly pass unrecorded. Nevertheless they are the true glories of war, before which the pomps of national triumph fade quite away. They show the martyrdom of the spirit before the savagery of blood and iron, the conquest of the soul over the weakness of the flesh. They bring a sure promise of the ultimate victory of the spirit, and of the immortality of the soul.

For Nursing Affairs in Ireland, see p. 74; Matrons' and Sisters' Appointments, p. 84.

